# The Gut Microbiota (Microbiome) in Cardiovascular Disease and Its Therapeutic Regulation

**DOI:** 10.3389/fcimb.2022.903570

**Published:** 2022-06-20

**Authors:** Md. Mominur Rahman, Fahadul Islam, Md. Harun -Or-Rashid, Abdullah Al Mamun, Md. Saidur Rahaman, Md. Mohaimenul Islam, Atkia Farzana Khan Meem, Popy Rani Sutradhar, Saikat Mitra, Anjuman Ara Mimi, Talha Bin Emran, Fatimawali, Rinaldi Idroes, Trina Ekawati Tallei, Muniruddin Ahmed, Simona Cavalu

**Affiliations:** ^1^ Department of Pharmacy, Faculty of Allied Health Sciences, Daffodil International University, Dhaka, Bangladesh; ^2^ Molecular Pharmacology Research Center, School of Pharmaceutical Sciences, Wenzhou Medical University, Wenzhou, China; ^3^ Department of Pharmacy, Faculty of Pharmacy, University of Dhaka, Dhaka, Bangladesh; ^4^ Department of Pharmacy, BGC Trust University Bangladesh, Chittagong, Bangladesh; ^5^ Pharmacy Study Program, Faculty of Mathematics and Natural Sciences, University of Sam Ratulangi, Manado, Indonesia; ^6^ Department of Pharmacy, Faculty of Mathematics and Natural Sciences, University of Syiah Kuala, Banda Aceh, Indonesia; ^7^ Department of Chemistry, Faculty of Mathematics and Natural Sciences, Universitas Syiah Kuala, Banda Aceh, Indonesia; ^8^ Department of Biology, Faculty of Mathematics and Natural Sciences, University of Sam Ratulangi, Manado, Indonesia; ^9^ Faculty of Medicine and Pharmacy, University of Oradea, Oradea, Romania

**Keywords:** gut microbiota, cardiovascular disease, metabolites, trimethylamine, atherosclerosis, hypertension

## Abstract

In the last two decades, considerable interest has been shown in understanding the development of the gut microbiota and its internal and external effects on the intestine, as well as the risk factors for cardiovascular diseases (CVDs) such as metabolic syndrome. The intestinal microbiota plays a pivotal role in human health and disease. Recent studies revealed that the gut microbiota can affect the host body. CVDs are a leading cause of morbidity and mortality, and patients favor death over chronic kidney disease. For the function of gut microbiota in the host, molecules have to penetrate the intestinal epithelium or the surface cells of the host. Gut microbiota can utilize trimethylamine, N-oxide, short-chain fatty acids, and primary and secondary bile acid pathways. By affecting these living cells, the gut microbiota can cause heart failure, atherosclerosis, hypertension, myocardial fibrosis, myocardial infarction, and coronary artery disease. Previous studies of the gut microbiota and its relation to stroke pathogenesis and its consequences can provide new therapeutic prospects. This review highlights the interplay between the microbiota and its metabolites and addresses related interventions for the treatment of CVDs.

## 1 Introduction

According to the World Health Organization (WHO), cardiovascular diseases (CVDs) cause the deaths of 17.9 million people per year, corresponding to 31% of all deaths. Of these, 85% are directly associated with stroke and heart attack. Arterial hypertension, coronary artery disease (CAD), and cardiomyopathies that reinforce heart failure and cerebrovascular diseases at the end phase are typically categorized as CVDs under non-communicable conditions (Velasquez et al., 2018; Marzullo et al., 2020). Dyslipidemia (i.e., elevated serum cholesterol, triglycerides, and low-density lipoproteins), hypertension, obesity, smoking, and diabetes are probable causes of atherosclerosis (Ussher et al., 2013). Current interventions for atherosclerosis that target these risk factors, such as first-line statins (which inhibit 3-hydroxy-3-methyl-glutaryl-CoA reductase and prevent it from decreasing the production of cholesterol) are beneficial in disease prevention and treatment. However, a substantial percentage of patients remain highly resistant to treatment with statins and other traditional treatments. Monitoring their atherosclerotic condition exacerbates and potentially causes other coronary disorders such as myocardial infarction (MI) or stroke (Davignon, 2004). Apart from genetic factors, environmental factors such as nutrition and intestinal microbiota composition are also considered significant for the development of CVDs. Additionally, intestinal dysbiosis, a key risk factor for CVDs, has been correlated with the development of obesity and diabetes (Almeida et al., 2021).

The human intestinal microbiota comprises more than 10 trillion microorganisms, including bacteria, archaea, viruses, protozoa, and fungi. A healthy microbiota consists primarily of four groups of bacteria, namely Actinobacteria, Firmicutes, Proteobacteria, and Bacteroides, and they continuously adapt these to lifestyle changes (Canfora et al., 2019). The human microbiome is the collection of all microorganisms occupying the body, also termed the microbiota (Rinninella et al., 2019). Determining the composition of a natural microbiome is a complex process that requires consideration of the functioning of the heart, the stable ecosystem of the environment, and the microbial ecology and associated metabolites from the tolerance, durability, and stability perspectives. Within the host, the distribution of microbes is relatively stable, with consistency among family members; however, the composition differs widely among unrelated individuals from different geographies (Lloyd-Price et al., 2016; Lloyd-Price et al., 2017).

The gut microbiota interacts with the host through the intestinal mucosal surface, and the function of the intestinal epithelial barrier is maintained through several functions of a well-balanced gut microbiota, such as the restoration of tight junction protein structure, mucin gene upregulation, and inhibition of epithelial cell binding with pathogenic bacteria. Intestinal wall edema may decrease intestinal blood flow in the context of compromised cardiac and/or renal function, which may occur due to the structural disruption of the mucosal epithelial barrier and increased permeability (Vaziri et al., 2012; Ramezani and Raj, 2014). Disruption of the intestinal wall facilitates the translocation of endotoxins, microbial elements, and microbial metabolites into the systemic circulation, which may trigger immune responses and amplify systemic inflammation. Circulating bacterial lipopolysaccharides (LPS) facilitate chronic kidney disease (CKD) and increase mortality risk. Additionally, bacterial DNA can be detected in the blood of patients with CVD and CKD (Dinakaran et al., 2014; Shi et al., 2014).

The synthesis of trimethylamine N-oxide (TMAO) and the development of cardiovascular risk exhibit another dimension to this dynamic activity, i.e., the interplay between the intestinal microbiome and the human host that occurs through the interaction of dietary intake (a type of environmental exposure) with the intestinal microbiota, leading to the production of metabolites that may serve as cardiac disease boosters (Tang et al., 2014a; Tang et al., 2014b). Given the high levels of production of trimethylamine (TMA) and TMAO by choline-induced gut microbiota, decreased intake of dietary TMAO precursors is a viable pathway to reducing the risk of CVD (Koeth et al., 2013; Wang et al., 2015).

This review highlights several recent advances in our understanding of the gut microbiota’s role in the development of atherosclerosis and associated severe CVD complications. It also addresses strategies for targeting the gut microbiota that contribute to the generation of TMAO for the potential prevention and treatment of CVD as well as the role of gut microbiota in CVD and potential corresponding interventions.

## 2 Molecular Functions of Gut Microbiota

The gut microbiota has several effects on the host (**Table 1**). The microbes send signals that must pass through the intestinal epithelium to multiple organs. These signaling molecules also form the basic structure of the microbiota and are made of LPS and peptidoglycans. They are mainly associated with host mucosal cell surfaces that contain pattern recognition receptors (PRRs; Larsson et al., 2012). These receptors’ primary function is to identify the pathogenicity of associated molecular patterns, by which they can provide instructions for stimulating the host immune system (Brown and Hazen, 2015; Cardos et al., 2021). The signaling process is induced by the recognition of LPS and peptidoglycans by host receptors situated in epithelial cells (Cani et al., 2007).

**Table 1:** Selected small molecules from the human gut microbiota with name, class, origin, and activities within human body (Donia and Fischbach, 2015).

SI	Compound	Class	Microorganism (Example)	Host Site	Known/Predicted Activity
1	indolepropionic acid	Amino acid metabolite	*Clostridium sporogenes*	Gut	Immunomodulatory
2	indole	Amino acid metabolite	Unknown	Gut	Converted to indoxylsulfate
3	skatole	Amino acid metabolite	*Clostridium* spp.	Gut	Unknown
4	tryptamine	Amino acid metabolite	*Ruminococcus gnavus*	Gut	Neurotransmitter
5	phenylacetic acid	Amino acid metabolite	*Bifidobacterium* spp.	Gut	Unknown
6	phenethylamine	Amino acid metabolite	*Lactobacillus* spp.	Gut	Neurotransmitter
7	δ-aminovaleric acid	Amino acid metabolite	*Clostridium* spp.	Gut	Unknown
8	GABA	Amino acid metabolite	Unknown	Gut	Unknown
9	α-aminobutyric acid	Amino acid metabolite	Unknown	Gut	Unknown
10	3-aminoisobutyric acid	Amino acid metabolite	*Clostridium* spp.	Gut	Unknown
11	*p*-cresol	Amino acid metabolite	*Clostridium* spp.	Gut	Unknown
12	lactocillin	RiPP (thiopeptide)	*Lactobacillus gasseri*	Vagina	Antibiotic
13	epidermin	RiPP (lantibiotic)	*Staphylococcus epidermidis*	Skin	Antibiotic
14	salivaricin A2 and B	RiPP (lantibiotic)	*Streptococcus salivarius*	Mouth	Antibiotic
15	cytolysin	RiPP (lantibiotic)	Enterococcus faecalis*	Gut	Antibiotic, Cytotoxic
16	ruminococcin A	RiPP (lantibiotic)	*Ruminococcusgnavus*	Gut	Antibiotic
17	staphylococcin Au-26 (Bsa)	RiPP (lantibiotic)	*Staphylococcus aureus*	Skin	Antibiotic
18	SA-FF22	RiPP (lantibiotic)	*Streptoccous pyogenes*	Oral/Skin	Antibiotic
19	ruminococcin C	RiPP(bacteriocin)	*Ruminococcus gnavus*	Gut	Antibiotic
20	microcin C7/C51	RiPP (microcin)	*Escherichia coli*	Gut	Antibiotic
21	microcin B17	RiPP (microcin)	*Escherichia coli*	Gut	Antibiotic
22	microcin J25	RiPP (microcin)	*Escherichia coli*	Gut	Antibiotic
23	microcin H47	RiPP (microcin)	*Escherichia coli*	Gut	Antibiotic
24	streptolysin S	RiPP (TOMM)	*Streptococcus pyogenes*	Oral/Skin	Cytotoxic
25	clostridiolysin S	RiPP (TOMM)	*Clostridium sporogenes*	Gut	Unknown
26	listeriolysin S	RiPP (TOMM)	*Listeria monocytogenes*	Gut	Unknown
27	heat-stable enterotoxin	RiPP	*Escherichia coli*	Gut	GI motility (guanylate cyclase 2C)
28	*p*-cresol	Amino acid metabolite	*Clostridium* spp.	Gut	Unknown
29	propionic acid	Acid (short-chain)	*Bacteroides* spp.	Gut	Immunomodulatory (GPR43)
30	polysaccharide A	Oligosaccharide	*Bacteroides fragilis*	Gut	Immunomodulatory (TLR2)
31	capsular polysaccharide	Oligosaccharide	*Streptococcus pneumoniae*	Airways	Immunomodulatory
32	α-galactosylceramide	Glycolipid	*Bacteroides fragilis*	Gut	Immunomodulatory (CD1d)
33	corynomycolic acid	Glycolipid	*Corynebacterium* spp.	Skin	Unknown
34	mycolic acid	Glycolipid	*Mycobacterium* spp.	Airways	Immunomodulatory (CD1b)
35	muramyl di- and tripeptides	Glycopeptide	*Fusobacterium nucleatum*	Oral	Immunomodulatory (NOD1, NOD2)
36	staphyloxanthin	Terpenoid	*Staphylococcus aureus*	Skin	Unknown (antioxidant)?
37	bile acids (e.g., deoxycholic acid)	Terpenoid	*Clostridium* spp.	Gut	Metabomodulatory (TGR5, FXR, VDR)
38	phevalin	NRP	*Staphylococcus aureus*	Skin	Unknown (virulence inducer)?
39	cereulide	NRP	Bacillus cereus*	Gut	Cytotoxic, Immunomodulatory
40	yersiniabactin	NRP	Yersinia pestis*	Bloodstream	Siderophore
41	cyanobactin	NRP	*Corynebacterium* spp.	Skin	Siderophore
42	tilivalline	NRP	Klebsiellaoxytoca*	Gut	Cytotoxic
43	zwittermicin	NRP-PK	Bacillus cereus*	Gut*	Antimicrobial
44	mutanobactin	NRP-PK	*Streptococcus mutans*	Mouth	Unknown
45	colibactin	NRP-PK	*Escherichia coli*	Gut	Cytotoxic
46	mycolactone	PK	Mycobacterium ulcerans*	Skin	Immunomodulatory
47	coproporphyrin III	Porphyrin	*Propionibacterium acnes*	Skin	Unknown
48	staphyloferrin B	Citrate amide	*Staphylococcus aureus*	Skin	Siderophore

The gut microbiota can directly or indirectly attack distant host organs. It also affects the bioactive metabolite system (Medzhitov, 2007; Islam et al., 2021) and mediates attacks on the host *via* the bile acid route and trimethylamine (Downes et al., 2003; Ma et al., 2006; Watanabe et al., 2006; Gao et al., 2009; Thomas et al., 2009; Pols et al., 2011; Wang et al., 2011a; Kimura et al., 2013; Koeth et al., 2013; Pluznick et al., 2013; Tang et al., 2013; De Vadder et al., 2014; Zhu et al., 2016). Moreover, the gut microbiota utilizes endocrine hormones, including leptin (Ryan et al., 2014; Tremaroli et al., 2015). Perry et al. (2016) showed that the PNS directly increases the metabolic functions of the host body by regulating the gut microbiota (Perry et al., 2016).

## 3 Mechanisms of Gut Microbiota Function in the Host

The gut microbiota is composed of colonies of bacteria, including intestinal *Bacteroidetes* (Eckburg et al., 2005). These bacteria are stably present at various sites of the gut despite being comprised of various species. Several gut microorganisms can be differentiated according to their location, mainly in the ascending colon (Eckburg et al., 2005). More than 90% of bacteria influence the growth of *Bacteroidetes* and *Firmicutes*, and the *Firmicutes*/*Bacteroidetes* (F/B) ratio remains the same in subjects with CVD (Gill et al., 2006). A report showed that Ukrainian adults had a high body mass index (BMI) even though their F/B ratio was the same in the absence of unhealthy habits such as smoking (Koliada et al., 2017). The F/B ratio becomes a harmful factor in children in the context of obesity (Indiani et al., 2018). Every rate has a low grade of inflammation that may result in diabetes (Pascale et al., 2019). This disease is a risk factor for CVDs. The gut microbiota communicates with the host to protect intestinal integrity because the gut behaves like a carrier among them (Zhou et al., 2020).

TMAO is a molecule formed by the digestion of choline-, lecithin-, and L-carnitine-containing foods, predominantly animal products, with a few plant-derived elements. The gut microbiota metabolizes lecithin (which includes phosphatidylcholine, a source of choline) and choline in foods into TMA. L-carnitine is thought to be converted to γ-butyrobetaine in a second step. TMA is oxidized into TMAO in the liver. According to new data, high circulating levels of TMAO are linked to an increased risk of CVD and mortality.

**Figure 1:**
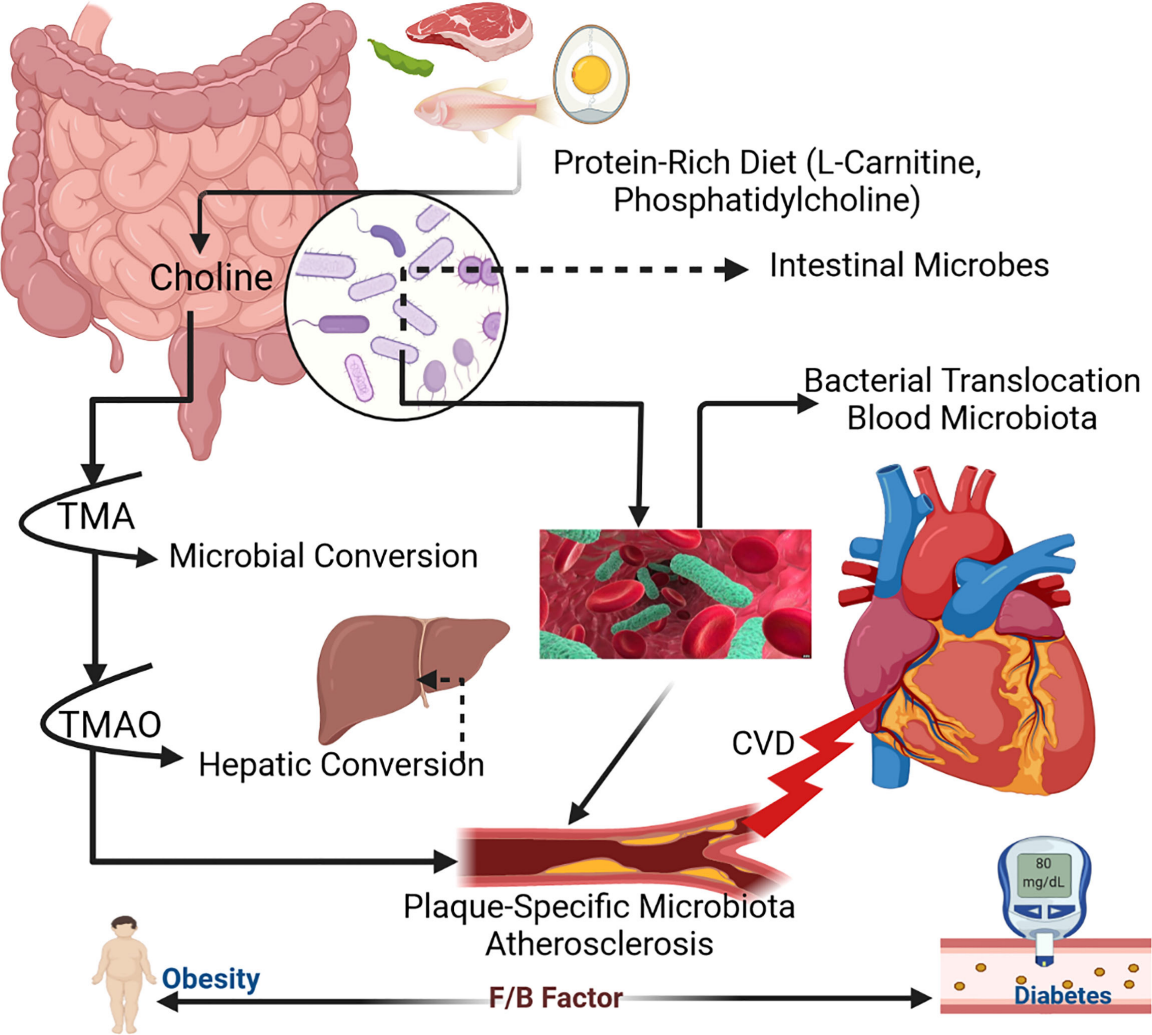
Mechanism of human gut microbiota within host body (Zhu et al., 2017).

Many variables contribute to this elevated risk, including changes in cholesterol and bile acid metabolism and the activation of inflammatory pathways. Higher levels of TMAO in the arteries increases cholesterol deposition from the circulation, contributing to atherosclerosis. The inflammatory reaction is a key contributor to the progression of renal disease and/or the development of metabolic syndrome and/or type 2 diabetes (**Figure 1**; Koeth et al., 2013; Tang et al., 2013; Koeth et al., 2014; Lemos et al., 2018).

## 4 Role of Gut Microbiota in the Host

Several types of microorganisms, including bacteria and viruses, are present in the body. Microbial colonies survive *via* coexistence with the host, collectively forming the microbiota. Several microorganisms colonize host organisms to survive. Generally, the microbiota that colonizes the gastrointestinal tract (GIT), specifically the colon, is termed the primary anaerobic microbiota. Nutrients are digested by colonies of microbes in the intestine by proteolytic and saccharolytic pathways (Sekirov et al., 2010). Through the saccharolytic pathway, the gut microbiota metabolizes small sugar chains, producing SCFAs. In the catabolic pathway, microbes use enzymes to ferment proteins, influence SCFA production, and increase metabolic sites like phenol. However, some metabolic processes result in toxicity due to renal excretion (Nallu et al., 2017). The gut microbiota forms a relationship with the host and increases digestive activity. It also controls the function of the intestinal mucosal layers and preserves nutrients and metabolism. Prior investigations have demonstrated that the gut microbiota provides immunological tissue and reduces the pathogenicity of microorganisms (Savage, 1970; Bäckhed et al., 2004; Bäckhed et al., 2005; Mazmanian et al., 2005; Li et al., 2008). Under suitable conditions, the gut microbiota influences the immune system so that the body can defend itself against pathogens (Rakoff-Nahoum et al., 2004).

By reducing the inhibition of lipoprotein lipase activity in adipocytes, the gut microbiota has been demonstrated to favorably influence lipid metabolism. Furthermore, Bacteroides taiotaomicron has been shown to improve lipid hydrolysis efficiency by upregulating the production of a colipase necessary for lipid digestion by pancreatic lipase (Hooper et al., 2001).

Another key metabolic function of the gut microbiota is the synthesis of vitamin K and various components of vitamin B. Members of the genus Bacteroides have been shown to produce conjugated linoleic acid (CLA), which is an anti-diabetic, antiatherogenic, anti-obesogenic, hypolipidemic, and immunomodulatory fatty acid (Devillard et al., 2007; Devillard et al., 2009; Baddini Feitoza et al., 2009). According to current data, the human gut microbiota is also involved in the degradation of numerous polyphenols (phenolic chemicals) absorbed from food. Flavanols, flavanones, flavan-3-ols, anthocyanidins, isoflavones, flavones, tannins, lignans, and chlorogenic acids are polyphenolic secondary metabolites present in a range of plants, fruits, and plant-derived products such as tea, chocolate, and wine (Marín et al., 2015; Cavalu et al., 2018; Miere et al., 2020; Mominur Rahman et al., 2021).

A healthy gut microbiota as required for optimal homeostasis places the gut mucosal immune system in a difficult position as it must tolerate beneficial commensals while preventing pathogen overgrowth. A two-tiered mucus layer, which keeps luminal microorganisms away from epithelial contact, is a fundamental mechanism of antimicrobial defense, especially in the large intestine. Mucus comprises a variety of mucin glycoproteins released by goblet cells in the intestine and can extend up to 150 meters from the colonic epithelium (Johansson et al., 2008).

The gut microbiota has been shown to promote the formation of antimicrobial proteins (AMP) such as cathelicidins, C-type lectins, and (pro)defensins by host Paneth cells through a PRR-based pathway involving its structural components and metabolites (Artis et al., 2004; Hooper, 2009). The PRR family (nucleotide-binding oligomerization domain-like receptors [NLRs]) contains membrane-associated Toll-like receptors (TLRs), C-type lectin receptors (CLRs) like Dectin-1, and cytosolic nucleotide-binding and oligomerization domain (NOD)-like receptors (Salzman et al., 2007; Takeuchi and Akira, 2010).

The gut microbiota has also evolved a strategy to prevent the overgrowth of pathogenic strains through the generation of local immunoglobulins. The gut microbiota, particularly Gram-negative bacteria such as Bacteroides, has been found to stimulate intestinal dendritic cells (DCs), causing plasma cells in the intestinal mucosa to secrete IgA (sIgA; (He et al., 2007).

In addition to the innate and adaptive immune systems, the gut microbiota plays a role in gut immunomodulation. The gut-associated lymphoid tissues (GALT), effector and regulatory T cells, IgA-producing B (plasma) cells, Group 3 innate lymphoid cells, and resident macrophages and DCs in the lamina propria are all immune system components and cell types that participate in immunomodulatory the process (Macpherson and Uhr, 2004).

## 5 Interventions

### 5.1 Dietary Interventions of Gut Microbiota

Many studies have found that dietary therapies can significantly lower cardiovascular risk (Cavalu et al., 2013; Sindhu et al., 2021). A Mediterranean diet has been found to reduce the prevalence of CVDs as well as mortality rates. Accumulating evidence suggests that dietary interventions can alter the influence of microbiota (Mekki et al., 2010; Ravussin et al., 2012; Estruch et al., 2013; David et al., 2014). Diet-dependent postprandial blood glucose levels were associated with human gut microbiota composition in a systematic analysis involving > 900 participants. Dietary interventions that regulate significant changes in these components can alter the composition and microenvironment of the microbiota (David et al., 2014; Voreades et al., 2014; Xu et al., 2015).

Variations in *Roseburia* and *E. rectale* were found to be associated with differences in the proportion of dietary carbohydrate content. The growth of beneficial commensal bacteria is promoted by fiber-rich diets and prevents the development of known opportunistic pathogens (Ley et al., 2006b; Duncan et al., 2007; Foye et al., 2012). A high-fiber diet has been reported to increase the proportion of acetate-producing microbiota, reduce blood pressure (BP), and alleviate heart hypertrophy and fibrosis (Marques et al., 2017).

### 5.2 Probiotic, Prebiotic, and Antibiotic Intervention

An adequate amount of probiotics was found to regulate obesity and hyperglycemia (Natarajan et al., 2014). The study confirmed that the administration of *Christensenellaminuta* altered microbial ecology and protected mice from obesity. Additionally, in a recent study, *Lactobacillus reuteri* administration was found to improve insulin secretion by encouraging incretin release in obese subjects (Simon et al., 2015). Similarly, the administration of *Lactobacillus* sp. in patients with CKD was correlated with a substantial decrease in toxins released by the small intestine, such as dimethylamine and nitroso dimethylamine, along with improvements in the colon levels of some short-chain fatty acids (SCFAs) in carotid atherosclerosis patients (Simenhoff et al., 1996; Karlsson et al., 2010).

Prebiotics are a group of non-digestible carbohydrates that selectively change the composition and activities of the microbiome. Recent data revealed that prebiotic foods such as dietary fibers, various oligo- and polysaccharides, and resistant starches preserve the balance of the gut microbiota (Roberfroid et al., 2010; Adamberg et al., 2018). Typical prebiotic molecules are indigestible food molecules such as oligosaccharides or complex saccharides. Several studies have suggested that the administration of prebiotics regulates glycemia and plasma lipid profiles. Specifically, 3 months of oligofructose supplementation was found to remarkably improve obesity, weight loss, and glucose tolerance (Robertson et al., 2005; Parnell and Reimer, 2009). In a preclinical analysis using an animal model of insulin resistance, antibiotics and prebiotics were reported to counteract microbial population characteristics associated with diabetes mellitus, increase gut permeability, decrease metabolic endotoxemia, suppress inflammation, and promote sugar intolerance (Everard et al., 2011). However, nonspecific antimicrobial interventions cannot substantially provide desired therapeutic outcomes. It links antibiotic use in humans with childhood obesity within the first 6 months of development (Trasande et al., 2013; Rahman et al., 2021b). Emerging evidence implied that treatment with vancomycin and minocycline decreased systolic BP in hypertensive rats. Moreover, ampicillin administration reduced atherosclerotic risk factors such as lipoprotein levels (Rune et al., 2016; Galla et al., 2018).

These findings show that preventive improvements in the gut’s microbial makeup may protect the beneficial microbiota essential for sustaining well-being since specific microbiota or their metabolites can induce defensive cardiovascular effects. Thus, individualized microbiota-based treatment programs can provide new therapeutic options for cardiometabolic disorders.

## 6 Pharmacology and the Microbiome

**Figure 2:**
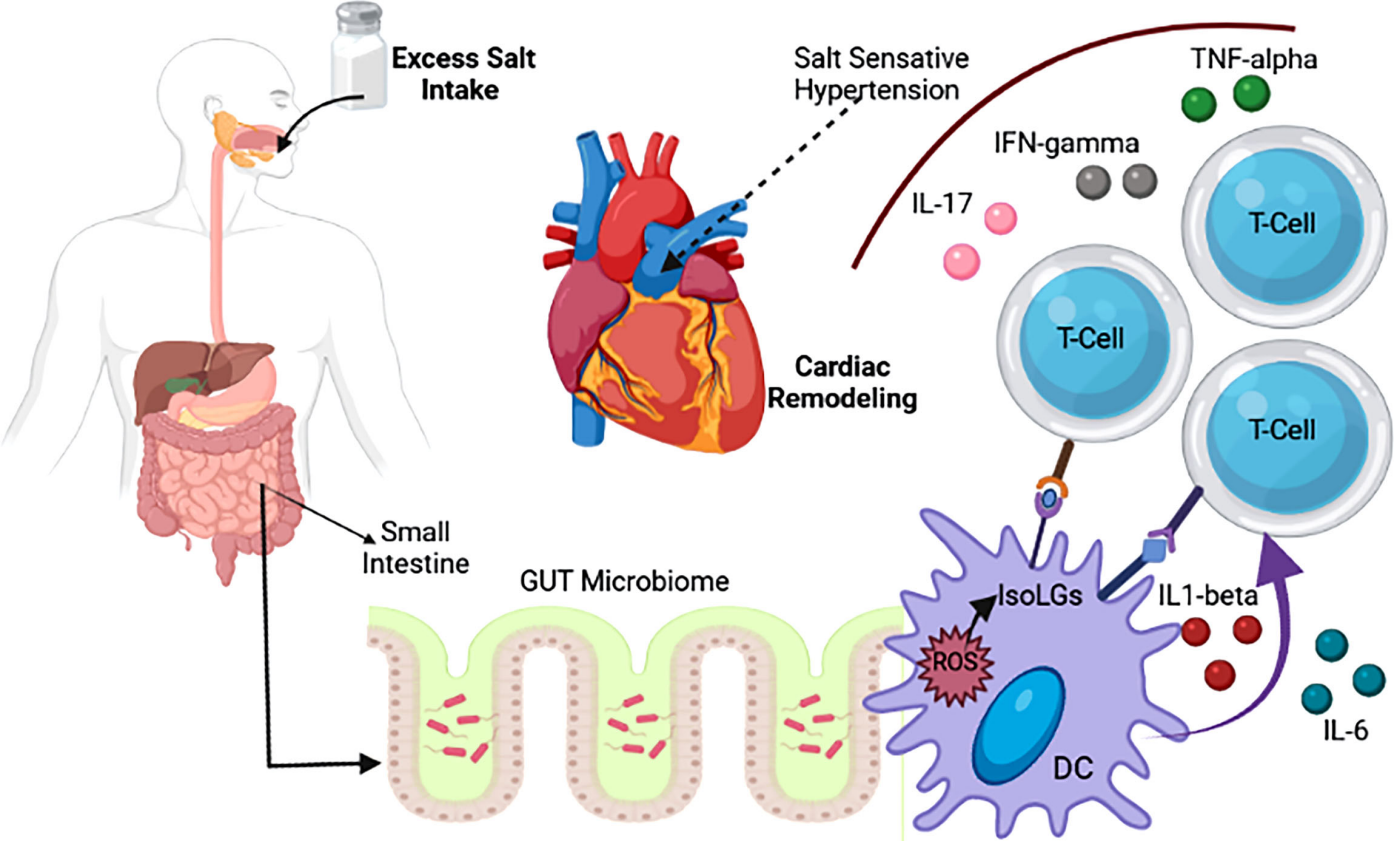
Association of salt intake with change in gut microbiotia and CVD (Naqvi et al., 2021).

Although evidence shows that changes in the microbiome may affect various disease pathologies such as diabetes, obesity, hypertension, and heart disease, the microbiota can also trigger drug responses (Clayton et al., 2009; Spanogiannopoulos et al., 2016). Different chemicals released by gut bacteria can interfere with a drug’s pharmacokinetics and pharmacodynamic effects (**Figure 2**; Elrakaiby et al., 2014; Sharma et al., 2019). While the liver is the primary site of drug metabolism, it has recently been discovered that almost all oral drugs affect the gut microbiome (Vich Vila et al., 2020). Individual therapeutic responses and drug-related side effects differ based on various factors such as gender, age, BMI, disease state, environmental factors, and genetic polymorphisms (Lammers et al., 2020).

In ampicillin-treated rats, Yoo et al. (2016) found that plasma concentrations of amlodipine, a calcium channel blocker used as a standard treatment for hypertension, increased by 133% (Yoo et al., 2016). For Ag-II-induced hypertension, sodium butyrate and aSCFA stimulants that work in tandem with angiotensin-converting enzyme (ACE) inhibitors have proven effective. Enalapril is a common therapeutic ACE blocker that can strengthen the intestinal barrier by stimulating gut perfusion (Kincaid et al., 1998; Pérez-Paramo et al., 2000).

Three recent investigations used different sequencing approaches to study the gut microbiota composition in individuals with CAD (**Table 2**). Cui et al. (2017) found phylum-level variations in the gut microbiota of individuals with CAD, with a lower proportion of *Bacteroidetes* and a higher proportion of *Firmicutes* (Cui et al., 2017). Jie et al. (2017) found elevated levels of various members of the genus Streptococcus and the Enterobacteriaceae family and decreased abundance of *Roseburia intestinalis* and *Faecalibacteriumprausnitzii*, both known producers of the SCFA butyrate (Jie et al., 2017). Escherichia-Shigella and Enterococcus were found to be more abundant, while the butyrate producers *Faecalibacterium*, *Roseburia*, and *Eubacterium rectale* were found to be less productive (Zhu et al., 2018). The findings of Jie et al. and Zhu et al. are consistent with a recent investigation of symptomatic carotid atherosclerosis by Karlsson et al. (2012), which found a lower relative abundance of *Roseburia* and *Eubacterium*, recognized butyrate producers (Karlsson et al., 2012). Butyrate and other SCFAs are the end products of dietary fiber fermentation and the primary energy source for colonocytes that preserve the gut mucosal barrier (Roediger, 1982).

**Table 2:** In individuals with coronary artery disease (CAD), modern gut microbiota sequencing investigations are being conducted.

Study	(Cui et al., 2017)	(Jie et al., 2017)	(Zhu et al., 2018)
Patients	CAD verified by coronary angiography	CAD verified by coronary angiography	CAD verified by coronary angiography
Patient age	68.3 ± 9.5 years	40-80 years	63.5
Gender% (f/m)	85/15	–	58/42
Sample size	n = 29 CAD n = 35 controls	n = 218 CAD n = 187 controls	n = 70 CAD n = 98 controls
Methods	16 s rRNA	Metagenomics	16 s rRNA
Parallel plasma/serum	No	No	No
Dietary data	No	No	No
Increased relative abundance in patients	-*Firmicutes* phylum	*Enterobacteriaceae* family and *Streptococcus* species	*Escherichia*-*Shigella* and *Enterococcus*
Decreased relative abundance in patients	*Bacteroidetes* phylum	*Roseburia Intestinalis* and *Faecalibacterium Prausnitzii*	*Faecalibacterium*, *Roseburia*, *Subdoligranulum* and *Eubacteriumrectale*.
Functional findings	–	-Less fermentative capacity and more inflammatory properties in CAD microbiomes	Several predicted functions, including lipopolysaccharide biosynthesis and propanoate metabolism enhanced in CAD microbiomes

Butyrate exerts local anti-inflammatory effects in the intestinal mucosa by activating colonic regulatory T cells; therefore, gut microbial alterations impacting butyrate production may also influence inflammatory pathways (Marques et al., 2017). A defective gut mucosal barrier may result from the loss of butyrate-producing bacteria, allowing the passive leakage of microbial toxins such as LPS, which bind to TLRs and other innate immune system receptors, triggering inflammation (Furusawa et al., 2013). It is worth noting that patients with CAD have microbiomes with a higher capacity for LPS production (Trøseid et al., 2013). LPS has been shown to have variable bioactivity, with hexa-acylated LPS inducing inflammation but not penta-acylated LPS (Brandsma et al., 2019).

The atherosclerotic process begins with a fatty streak and progresses to plaque rupture and acute atherothrombosis, which can lead to critical clinical events including stroke or myocardial infarction (Awoyemi et al., 2019). While inflammation plays a role at all stages of atherosclerosis, most of the research regarding the microbiome in CAD does not distinguish between chronic and acute events. The published investigations on the involvement of the microbiota in CAD have used cross-sectional designs to study patients with generally stable CAD (Brix et al., 2015; Vatanen et al., 2016). Thus, research on the microbiome during acute coronary syndromes and prospective studies powered by clinical events should be prioritized.

Several sequencing-based studies published in the last 2 years have found that the makeup and functions of the gut microbiota vary between patients with heart failure (HF) and healthy participants, with some consistent findings but significant variance between investigations (**Table 3**).

**Table 3:** Recent gut microbiome sequencing investigations in heart failure patients (HFP).

Study	(Luedde et al., 2017)	(Kamo et al., 2017)	(Kamo et al., 2017)	(Kummen et al., 2018; Mayerhofer et al., 2020)
Patients	Chronic HF: 70% exacerbation, 30% stable	Acute HF or exacerbation of chronic HF	Stable chronic HF: Ischaemic or dilated cardiomyopathy	Stable systolic HF
Age patients	65 ± 3.2 years	Two strata: 47.4 ± 2.8 years 73.8 ± 2.8 years	58.1 ± 13.3 years	58.9 (39-74) years
Gender% (f/m)	45/55	18/82	17/83	17/83
Sample size	n = 20 HF n = 20 controls	n = 12 HF <60years n = 10 HF >60years	n = 53 HF n = 41 controls	n = 84 HF (discovery- validation) n = 266 controls
Methods	16 s rRNA	16 s rRNA	16 s rRNA	16 s rRNA
Parallel plasma/serum	No	No	Yes	Yes
Dietary data	No	No	No	Yes
Increased relative abundance in patients	–	–	Ruminococcus gnavus	Prevotella, Hungatella, Succinclasticum
Decreased relative abundance in patients	*Coriobacteriaceae*, *Erysipelotrichaceae*, *Ruminococcaceae* (family level) *Blautia* (genus level)	-*Eubacteriumrectale*, *Dorealongicatena* -Depletion of *Faecalibacterium* in older patient	*Faecalibacterium Prausnitzii*	-*Lachnospiracea* family: 9 different genera, including *Blautia* and *Eubacteriumhalli* - *Ruminococcaceae*: *Faecalibacterium* -*Bifidobacteriaceae*: *Bifidobacterium*
Functional findings			- In HF microbiomes, increased capability for lipopolysaccharide biosynthesis and TMA generation, and decreased capacity for butyrate production.	- Butyrate production genetic potential is lower in HF microbiomes. - The bacterium *Eubacteriumhalli* is linked to soluble CD25 and mortality. - Dysbiosis linked to dietary fiber consumption

## 7 The Gut Microbiota as a Therapeutic Target

Current data on the gut microbiota and its connection to disease pathogenesis show potential for its therapeutic targeting. Drugs targeting the microbiome are still far from being manufactured but newly developed drugs provide opportunities for the treatment of stroke. Previous publications reported that TMAO was inhibited by antibiotic use, whose administration over an extended period has negative effects such as the development of clostridium colitis (Nie et al., 2018). However, recent studies have shown that the improvement of inhibition depends on TMAO, which should be able to minimize the risk of thrombosis (Roberts et al., 2018). CutC/D is an effective TMAO inhibitor in animals, decreasing the amount of plasma TMAO within 3 days and increasing platelet levels and thrombus formation without the risk of toxicity (Brandt et al., 2012). For the treatment of Clostridium infection, fecal microbiota is used broadly for the secure treatment of general stool (Brandt et al., 2012). In the case of animals, microbial colonies reduce stroke-related problems (Yin et al., 2015; Yamashiro et al., 2017). Probiotics can help the gut microbiome if cytokine modulation helps respond to neuroinflammation. It can function as a therapeutic compound to reduce stroke problems (Di Giacinto et al., 2005; Divyashri et al., 2015).

## 8 Keeping Your Intestinal Flora Unaltered: A Preventive Approach to Reducing Cardiovascular Risk Factors

**Figure 3:**
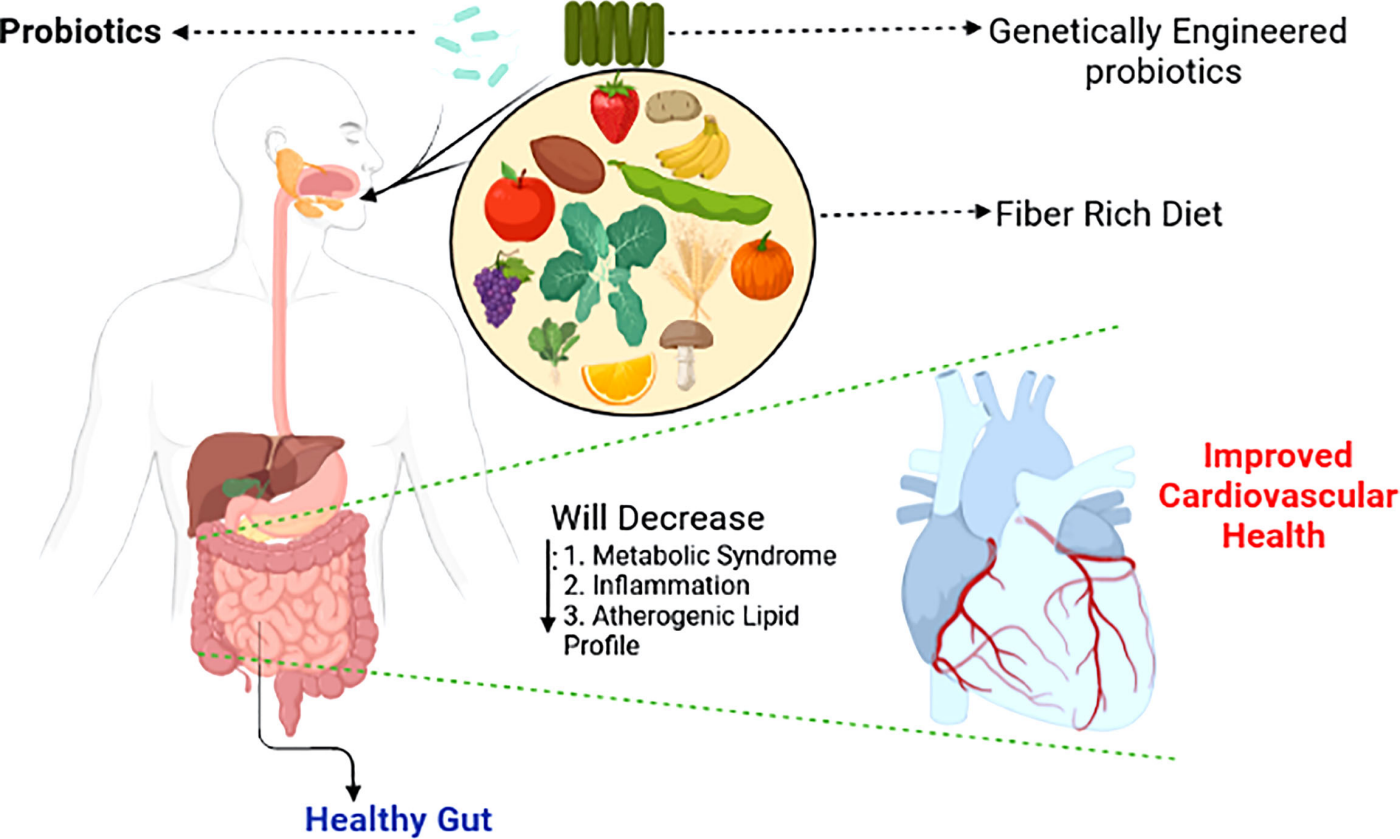
Shaping the gut microbiotia for cardiovascular benefits. Selective enrichment, using prebiotics and probiotics of beneficial bacteria alleviates major risk factors of cardiovascular disease (Singh et al., 2016).

Although therapeutic alterations in gut microbiota composition can improve host well-being, significant abrupt changes within the gut milieu through the use of antibiotics in humans during the first 6 months of life is associated with childhood obesity (Trasande et al., 2013). Similar results were observed in mice (Cho et al., 2012). Surprisingly, ApoE-KO mice fed a standard low-cholesterol diet and maintained under germ-free conditions develop severe atherosclerosis compared to their conventionally housed counterparts, suggesting that microbiota or their metabolites also mediate protective effects against CVD. A summary of therapeutic approaches that can be employed to alleviate CVD is shown in the schematic diagram (**Figure 3** **;** Stepankova et al., 2010).

## 9 Microbiota-Generated SCFA and Metabolites and CVD

### 9.1 Blood Pressure Regulation

By modifying the SCFAs of diastolic and systolic BP, CVD risk can be reduced and BP controlled. Testing in humans revealed that butyrate works as a helper in lowering diastolic BP (DPB; Roshanravan et al., 2017). During early pregnancy, bacteria produce a large amount of butyrate, which helps in lowering BP (Gomez-Arango et al., 2016). A meta-analysis revealed that all types of fiber help decrease BP, such as −.9 for systolic blood pressure (SBP) and −.7 for DBP. The range of this outcome was extensive in the case of beta-glucan-group fibers, being −2.9 for SBP and −1.5 for DBP (Evans et al., 2015).

### 9.2 Metabolic Regulation

A place for SCFA where it has more effect on the metabolic system. The relationship between strong animal and epidemiological shows opposite when compared to weight and dietary fiber. Recent studies have provided more knowledge about the role of SCFAs and found that acetate accelerates the production rate and regulates central appetite (Frost et al., 2014). This process takes place in the distal colon, which is more workable than the proximal colon, influencing oxidation of HAT, developing glucose levels, and alleviating inflammation (Overby and Ferguson, 2021). Acetate affects energy according to a recent review (Canfora and Blaak, 2017). Oral propionate works on humans as an accelerator, influencing oxidation rate (Chambers et al., 2018). In the case of animals, butyrate is effective orally but not intravenously. It decreases the rate of food intake but increases lipid and glucose levels through the neural circuit of the brain (gut; Li et al., 2018).

### 9.3 Gut Microbial Function

Soluble gut fiber decreases the rates of TMAO and TMA metabolism to 40.6% (TMA) and 62.6% (TMAO); it is significantly related to SCFA production and decreased serum lipids (Li et al., 2017). On the other hand, insulin supplementation did not affect starvation time (Chen et al., 2017).

#### 9.3.1 Function of the Gut Barrier

SCFAs and butyrate are considered critical substances for the maintenance of a healthy gut. Colonocytes prefer butyrate as a food source. SCFAs have recently been proposed to have a function in the control of epithelial integrity *via* the coordinated modulation of tight junction proteins that govern the intracellular molecular pathways between the lumen and the hepatic portal system. The movement of bacteria and/or their cell wall components is linked to hyperglycemia and increased gut permeability, which triggers an inflammatory process linked to obesity and insulin resistance (Thaiss et al., 2018). Butyrate can control significant components of the tight junction complex in mice by acting on NLRs, essential modulators of inflammation, in an FFAR2-dependent way (Cheng et al., 2018). Butyrate modulation of claudin proteins through p38 MAPK, IL-10 receptor mediation (Huang et al., 2014), and AMPK/intracellular ATP (Zheng et al., 2017) has been observed in cell models. Surprisingly, the inclusion of FFAR2 in this pathway suggests that other SCFAs may have a role in regulating barrier function (Yan and Ajuwon, 2017). Butyrate was shown to decrease steatohepatitis in a high-fat diet-induced steatohepatitis mouse model by repairing high-fat diet-induced damage to the intestinal mucosa, upregulating zonulin, and lowering endotoxin levels (Tong et al., 2016).

Downregulation of endotoxin-associated genes (TLR-4 and Myd88) and expression of proinflammatory genes in the liver were linked to these benefits. Given the growing importance of maintaining a functional physical barrier between luminal bacteria and the host immune system in the gut, well-designed studies in humans are needed to better understand SCFA’s involvement in gut barrier function.

#### 9.3.2 Energy Intake and Appetite Regulation

SCFAs have been linked to a reduction in appetite and calorie intake, which may protect against diet-induced obesity. Incorporating SCFAs into the diet of rats, on the other hand, has been shown in many experiments to have no influence on food consumption (De Vadder et al., 2014; Besten et al., 2015). Similarly, directly adding propionate into the diet had no effect on calorie intake during an ad libitum test meal or 24 hours after food consumption (Darzi et al., 2012). In recent research, Li et al. (2018) observed a decrease in energy intake after intragastric butyrate delivery but not after intravenous butyrate administration (Li et al., 2018).

Oral SCFA supplementation does not affect appetite responses according to the existing research in rats and humans, but administering SCFAs to a more distant location in the gut may lower energy intake. Because the SCFA receptors FFAR2 and FFAR3 are co-expressed in cells that express glucagon-like peptide 1 (GLP-1) and peptide YY (PYY), SCFAs may lower energy intake by promoting the production of these anorectic hormones (Kimura et al., 2014). This impact of SCFAs on gut hormone release has been studied extensively utilizing *in vitro* models of enteroendocrine cell lines (Psichas et al., 2014).

SCFAs can promote anorectic gut hormone secretion *via* FFAR2 according to these studies. High levels of SCFAs in the lower intestine have also been hypothesized to control energy intake *via* gut-brain neuronal pathways. For example, De Vadder et al. (2014) found that increased colonic propionate synthesis could trigger vagal signaling in the gut or portal vein *via* FFAR3 (De Vadder et al., 2014). Similarly, Li et al. (2018) discovered that after vagotomy, reduced food intake caused by the intragastric injection of butyrate in mice was prevented (Li et al., 2018).

### 9.4 Gut-Renal Axis and Uremic Toxicity

The intestinal tract contains many microbes, mainly in the form of colonies in the intestine (Nicholson et al., 2012). Poesen et al. (2016) tested two types of fecal samples from uremic and healthy patients (Poesen et al., 2016). It was also found that CKD was related to individual colonial macrobiotic mechanisms; despite the outcome, renal function decreases gradually, which may be less than the effect of diet and other factors associated with CKD. Misappropriation of ruined protein of the small intestine influences the consumption of dietary fiber (Kalantar-Zadeh et al., 2002).

They found para cresol (p-cresol) in urine, which was secreted for 24 hours. This component is responsible for the high levels of protein fermentation found in CKD (Aronov et al., 2011). Uremic toxin is produced in the human body mainly for microbial colonies. Aronov et al. (2011) tested the plasma of hemodialysis patients with colon and expected colon. There is a vast amount of p-cresol sulfate and IS in the human body (Vanholder et al., 2014). Additionally, the toxic level of p-cresol sulfide has been demonstrated previously (Karlsson et al., 2012). The pathogenic mechanisms of gut microbiota and metabolites in cardiometabolic diseases are shown in **Table 4**.

**Table 4:** Pathogenic mechanism of gut microbiota and metabolites in cardiometabolic diseases.

Category	Alterations in gut microbiota composition	Alterations in gut microbiota metabolites	Proof of concept	Interventions	References
**Atherosclerosis**	Increase *Lactobacillus*	Increase TMAO	Increased TMAO levels are linked to plaque instability and MACE (major adverse cardiac event)	Diet intervention: ↑Bactericides, Proteobacteria,	(Wang et al., 2011b; Koeth et al., 2013; Tang et al., 2013; Gan et al., 2014; Tachon et al., 2014; Gregory et al., 2015; Yang et al., 2015; Fu et al., 2016; Suzuki et al., 2017; Yamashiro et al., 2017)
	Decrease *Roseburiam*				
				Decrease Firmicutes Probiotics:	
			Increase TMAO DMB (1,3 dimethyl butanol)-microbial choline TMA lyase inhibition suppress TMA/TMAO		
				Increase LV (left ventricular) hypertrophy	
				Probiotics: Increase SCFA	
**Hypertension**	Increase *Firmicutes*/*Bacteroides* ratio	Increase SCFA	Infusion of Ang II (angiotensin II)/TMAO associated with BP	Diet intervention: a high-fiber diet is linked to lower BP	(Pluznick, 2013; Ufnal et al., 2014; Marques et al., 2017)
**Heart Failure**	Increase *Escherichia coli*, Klebsiella pneumonia, *Streptococcus viridians*	Increase TMAO	Increase Gut permeability	Diet: Eating a high-fiber diet has been linked to a reduction in heart hypertrophy and fibrosis	(Vaziri et al., 2013; Gan et al., 2014; Tang et al., 2015; Organ et al., 2016; Pasini et al., 2016)
			TMAO increase was linked to LV remodeling and poor prognosis		
				Probiotics: attenuate heart failure after myocardial infarction	
**Chronic Kidney Disease**	Increase *Firmicutes*, *proteobacteria*, and *actinobacteria*	Increase Indoxyl sulfate, p-cresol sulfate.	Ammonia disrupt the gut epithelial tight junction	Probiotics: decrease dimethylamine and nitroso dimethylamine	(Walsert and Bodenlost; Lin et al., 2011; Wu et al., 2011; Vaziri et al., 2013; Watanabe et al., 2013; Tang et al., 2014b; Tang et al., 2015; Organ et al., 2016)

In modern societies, CVD remains the leading cause of mortality (Graham et al., 2007). A study was performed using shotgun sequencing of the gut metagenome in Sweden and revealed the presence of the genus *Collinsella* in a large number of patients with symptoms of atherosclerosis. On the other hand, *Eubacterium* and *Roseburia* were abundant in healthy individuals (Karlsson et al., 2012).

A report on altered microbiota composition was published by a metagenome-wide organization (Jie et al., 2017) that examined 405 fecal samples of Chinese citizens. Of the subjects, 218 people had atherosclerosis CVD and the remaining 187 citizens were healthy. Individuals with atherosclerosis CVD were infected with *Enterobacteriaceae* and *Streptococcus* spp., and researchers determined the function of the gut microbiome from their stool samples. For example, these microbiomes had a higher potentiality regarding the shifting of simple sugars and amino acids (Karlsson et al., 2012).

### 9.5 Gut Microbial Interactions With Atherosclerosis and Heart Failure

Bacterial DNA is present in atherosclerotic plaques (Koren et al., 2011; Lins et al., 2013). These microbial colonies, which are made of bacteria, create and stabilize plaques, causing CVD. In humans, the oral microbiota has taxonomic characteristics that have also been discovered (Koren et al., 2011).

There are many epidemiological interrelations between CVD and periodontal disease (Mattila et al., 1989; Hyvärinen et al., 2012; Karlsson et al., 2012; Fåk et al., 2015). The oral microbiota displays pathogenic behavior in the case of CVD. Metagenomic sequencing of stool microbiota revealed that microbial composition is altered in people with unbalanced plaques. Moreover, these plaques relate to the decreased fecal level of the *Roseburiam* genus, and they both help increase the strength of the microbiota (Karlsson et al., 2012). The gut microbiota is a crucial contributor to the pathogenesis of heart failure. This hypothesis exposes decreasing cardiac output and increases the percentage of systemic circulation coagulation, which occurs in intestinal mucosal edema and ischemia and helps bacterial transport, increasing the endotoxin levels in the blood and thereby causing heart failure (Sandek et al., 2007).

A study reported that individuals with heart defects have high levels of endotoxin in the blood with edema and higher levels of proinflammatory components like cytokines than people without edema (Niebauer et al., 1999). Moreover, there is an interrelationship between CVD and TMAO. TMAO levels influence heart failure (Neish, 2009; Wang et al., 2011b; Tang et al., 2014a; Wang et al., 2014).

**Figure 4:**
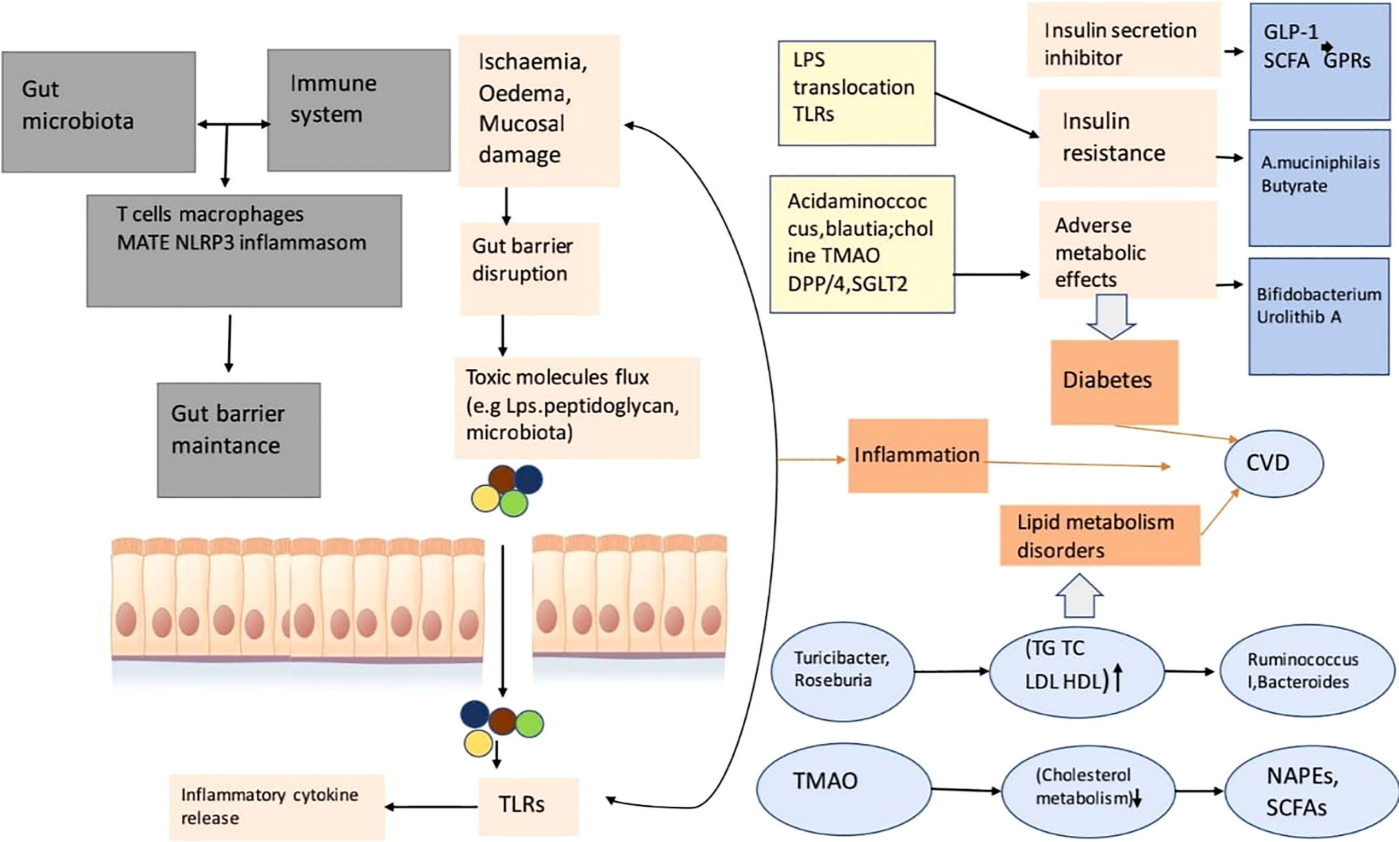
This is a diagram of cardiovascular risk and related to inflammation, the disorder of lipid metabolizing, diabetes relation with gut microbial disease. This gut microbiota reacts with the immune system of the animal or human body so that it can take control of the function of the gut barrier. A porous barrier influences the uprising of the flux of pro-inflammatory microorganisms (bacteria) into systemic circulation, thus the situation causes low-level inflammation through TLR activation (Xu et al., 2020).

High levels of TMAO are found in people with heart problems compared to healthy subjects (Tang et al., 2014a). By narrowing the wall of the heart with attenuated compression, a fraction of the heart is needed to dilate its chambers, a function found in mice that were fed a choline-containing diet. Changing growth element B phospho – SMAD3 route was found, which influences the diet of choline mice (**Figure 4** **;** Tang et al., 2014b).

### 9.6 The Intestinal Microbiome in Hypertension

Hypertension is a common but treatable cause of CVD. Studies have shown that the gut microbiota has a relationship with hypertension in humans, although rats exhibit high BP even without microbial colonization (Honour, 1982). However, now, a few studies have revealed a visible relationship between high BP and the gut microbiota in animals (Mell et al., 2015; Qi et al., 2015; Yang et al., 2015; Adnan et al., 2017). Yang et al. (2015) researched two types of rats. One was suffering from hypertension, and another had angiotensinogen 2 (chronic). They found an imbalance in the rats in that the overall number of microbes decreased and the number of Bacteroides was high in the hypertensive subjects (Yang et al., 2015). The presence of high numbers of microbes that produce butyrate lowers BP in overweight people and obesity in pregnant women (Mell et al., 2015). A growing body of research has reported that the gut microbiota directly regulates hypertension (Kawase et al., 2000; Tanida et al., 2005; Gómez-Guzmán et al., 2015).

### 9.7 Gut Microbiota and Uremic Toxins and CVD

Uremic toxicity is a big influencer of CVD risk related to CKD. Clinical studies have shown a relationship between CVD and CKD by testing individuals with and without CKD (Lin et al., 2010; Meijers et al., 2010; Lin et al., 2012; Wu et al., 2012; Hsu et al., 2013; Melamed et al., 2013; Sato et al., 2013; Shimazu et al., 2013; Cao et al., 2015; Lin et al., 2015; Shafi et al., 2015; Tsai et al., 2015; Yang et al., 2015).

Sato et al. (Shafi et al., 2015) surveyed the indoxyl sulfate (IS) levels of coronary artery patients and their estimated glomerular filtration rate was 60 mL/min/1.73 m^2^. Shimazu et al. (2013) observed an increased risk of hospital admission in cases of heart failure and in patients with CKD. They found that the cardiac disorder was associated with high levels of IS. IS levels were found to be directly related to higher rates of coronary artery calcification and cardiac drug-removing stent re-stenosis (Hsu et al., 2013; Tsai et al., 2015). Lin et al. (2012) reported that high levels of IS increased the risk of cardiovascular events but did not observe deaths among people with CKD (stage 3 to 5; Lin et al., 2015).

Nearly 50 years ago, Mitch (1978) drew attention to the value of intestinal microbiota in individuals with CKD. He studied nitrogen balance in uremic patients before and at the time of aminoglycoside antibiotic intake and observed that nitrogen from urea was not used by people with uremia in case of amino acid conjugation. Thus, the adverse effects of nitrogen balance improved (Aronov et al., 2011).

### 9.8 Gut Microbiota and Its Metabolism in CKD

Two types of changes occur in the intestinal microflora (dysbiosis) in the primary stages of CKD, one quantitative and the other qualitative. Thus, its formation and metabolic activities alter the microflora in CKD, making this a topic of interest in the field of nephrology. These changes include intestinal conduction and consumed protein absorption, reduced dietary fiber intake, oral iron treatment, and heavy use of antibiotics (Guldris et al., 2017).

Combustibility and uremic toxins play a leading role in the pathophysiology of atherosclerosis and other complications involved in CKD (Aron-Wisnewsky and Clément, 2016). The involvement of intestinal microbiota with the complex network of human organs is mediated by microbial metabolism in CKD, and intestinal-cardiac as well as intestinal-brain axes increase cardiovascular risk and may play a role in neuropsychiatric diseases (Cosola et al., 2019).

### 9.9 Kidney Crosstalk and Inflammation in the Intestine Due to CKD Development

Kidney crosstalk in the intestine, which is affected by the intestinal microbiota in most cases, plays an integral role in CKD development (Khoury et al., 2017). The intestinal microbiota mediate inflammatory (Khoury et al., 2017), neurological (Tanida et al., 2005; Rahman et al., 2020; Rahman et al., 2021a; Bhattacharya et al., 2022; Mominur Rahman et al., 2022), and endocrine (Afsar et al., 2016) processes in CKD. The microbiota protects gut health and CKD contributes to intestinal dysbiosis (Mahmoodpoor et al., 2017; Rahman et al., 2022b). CKD is associated with changes in the intestinal microbiota; species that produce uremic toxins, such as *Enterobacteria*, *Clostridiaceae*, *Pseudomonadaceae*, and Bacteroides increase in number, while beneficial species such as *Lactobacillus*, *Bifidobacteria*, and privateness decreased (Felizardo et al., 2016; Sampaio-Maia et al., 2016; Evenepoel et al., 2017; Kikuchi et al., 2017). Consequently, stool replacement in antibiotic-treated patients from CKD patient’s rats increased plasma TMAO levels (Xu et al., 2017).

### 9.10 Gut Microbiota and Energy Balance

The prevalence of obesity and related disorders is increasing globally. Although genetic susceptibility plays a significant role, much of this increase in obesity has occurred due to substantial changes in lifestyle over the past decade. The main factors include spending on energy-rich diets, lack of physical activity, a sedentary lifestyle, and unhealthy eating habits (Kushner and Choi, 2010). Intestinal microbiota were also identified as a factor involved in obesity development (Bäckhed et al., 2004). This concept has been built upon two observations: (1) the intestinal microbiota is distinct in non-obese and obese humans and rats (Ley et al., 2006b; Turnbaugh et al., 2008) and (2) the coarse phenotypes can be transferred by transferring microbiota from obese rats or human subjects to germ-free recipient rats (Turnbaugh et al., 2006; Ridaura et al., 2013).

### 9.11 Microbiota and Immunity

Early-life colon colonization of mammals plays an essential role in the host’s immune system (Gensollen et al., 2016). Host immunity can influence the most critical events in the first years of life, including the composition of the microbiota, which displays maximum intra- and inter-individual variability before reaching a stable configuration at 3 years of age (Koenig et al., 2011; Yatsunenko et al., 2012; Bäckhed et al., 2015). Increased susceptibility to various infectious pathogens characterizes the newborn’s immune system (Zhang et al., 2017). Moreover, an increased tendency toward excessive inflammation is frequent, as with babies born prematurely with the potential to develop the destructive disorder necrotizing enterocolitis (Neu and Walker, 2011).

## 10 Anti-Atherosclerotic and Anti-Cancer Effects of the Gut Microbiota

Atherosclerotic plaque, also known as atherosclerosis, is a localized lipid buildup in the artery wall that lowers blood vessel volume and can lead to hazardous thrombotic events. Low-density lipoprotein (LDL) is the primary cause of lipid buildup in atherosclerotic lesions. Consequently, a change in lipid metabolism favoring increased LDL and decreased high-density lipoprotein (HDL) levels, which aids cholesterol efflux and prevents its buildup, is an essential pathophysiological component of atherosclerosis progression. Additionally, chronic inflammation is a factor that has an equal, if not greater, role in the development of this disease (Geovanini and Libby, 2018).

A wealth of information suggests that mitochondrial damage, which causes oxidative stress and local inflammatory responses, plays a role in the initiation and development of atherosclerosis (Orekhov et al., 2019). Despite the diversity of etiology, clinical presentation, and treatment susceptibility, human malignancies share several essential characteristics that deliver promise for the development and enhancement of anti-cancer drugs. The inflammatory response is one such commonality since it has been demonstrated to play a key role in the development of a large variety of cancers (Diakos et al., 2014; Rauf et al., 2021; Rahman et al., 2022c). Mitochondrial dysfunction, increased reactive oxygen species (ROS) production, and the release of damage-associated molecular patterns (DAMPs) have all been linked to human cancer development (Yang et al., 2016 **;** Grazioli and Pugin, 2018).

Remarkably, information has been gained that relates the dysbiosis of the gut microbiota to the pathways behind insulin resistance, lipogenesis, fat accumulation, mitochondrial dysfunction, and systemic or local inflammation (Rath et al., 2018; Tang et al., 2019). Interactions between gut bacteria and intestinal epithelial surfaces of host cells have been identified to activate a variety of signaling pathways that regulate host pathophysiological processes such as energy metabolism, local and systemic inflammation, and oxidative stress (Campbell and Colgan, 2018). The molecular processes that support host-microbe contact and the accompanying pathophysiological reactions are complicated, and many are unknown. The production of intestinal GLP-1, regulation of hepatic SREBPs, activation of local or systemic Th17 cells and production of proinflammatory cytokines (IL-1, IL-6, TNF-a, and others), and induction of ROS have all been proposed in the last decade as plausible explanations for effects on energy balance, inflammation, and mitochondrial dysfunction (Cani and Jordan, 2018). In general, the binding of various recognized microbial components to cell receptors, such as LPS/TLR-4, peptidoglycan/NLRs, and flagellin/TLR-5, induces bacteria-directed innate pro-inflammatory pathways (Abreu, 2010). Anti-glycemic and anti-lipogenic pathways, for example, are thought to be helpful to the host in the fight against metabolic disorders. The creation of GLP-1 as a downstream signal from SCFA binding to G-protein-coupled receptor 41 (GPR41), GPR 43, and GPR 109A on the surface of the intestinal epithelium may be connected to these beneficial metabolic pathways (Canfora et al., 2019).

SCFAs can also cross the gut barrier and attach to receptors on other cell types, such as hepatocytes, adipocytes, and muscle cells, activating the AMPK pathway and improving glucose and lipid metabolism. Such metabolic outcomes of the interactions between the gut microbiota and the host appear to be significant not only in the pathogenesis of CVD but also in cancer biology. In fact, the metabolic condition of the tumor microenvironment can influence the anti-cancer immunity of tumor-infiltrating lymphocytes (TILs; O’Sullivan et al., 2019). For example, a new study found that metformin combined with immune checkpoint blockade (ICB) can improve clinical outcomes by increasing TIL anti-cancer activity and altering tumor metabolism (Eikawa et al., 2015 **)**

These processes are likely based on AMPK-dependent phosphorylation boosting PD-L1 degradation and AMPK-dependent suppression of tumor cell oxygen consumption (Foretz et al., 2014). Furthermore, patients with lung and kidney malignancies who had a poor response to PD-1 blocking had a decreased proportion of *Akkermansiamuciniphila* in their stomach, according to studies (Routy et al., 2018). The findings align with previous research linking gut bacteria to systemic and anti-tumor immunity in melanoma patients. Gut microbiome modulates response to anti–pd-1 immunotherapy in melanoma patients (Matson et al., 2018). These results confirm the gut microbiota’s function in modifying the anti-cancer benefits of various medications. They suggest that the etiology of atherosclerosis, metabolic syndrome, and cancer may share molecular pathways. Changes in the gut microbiota are a part of these pathways, making them a target for the development of new anti-atherosclerosis and anti-cancer medications.

**Figure 5:**
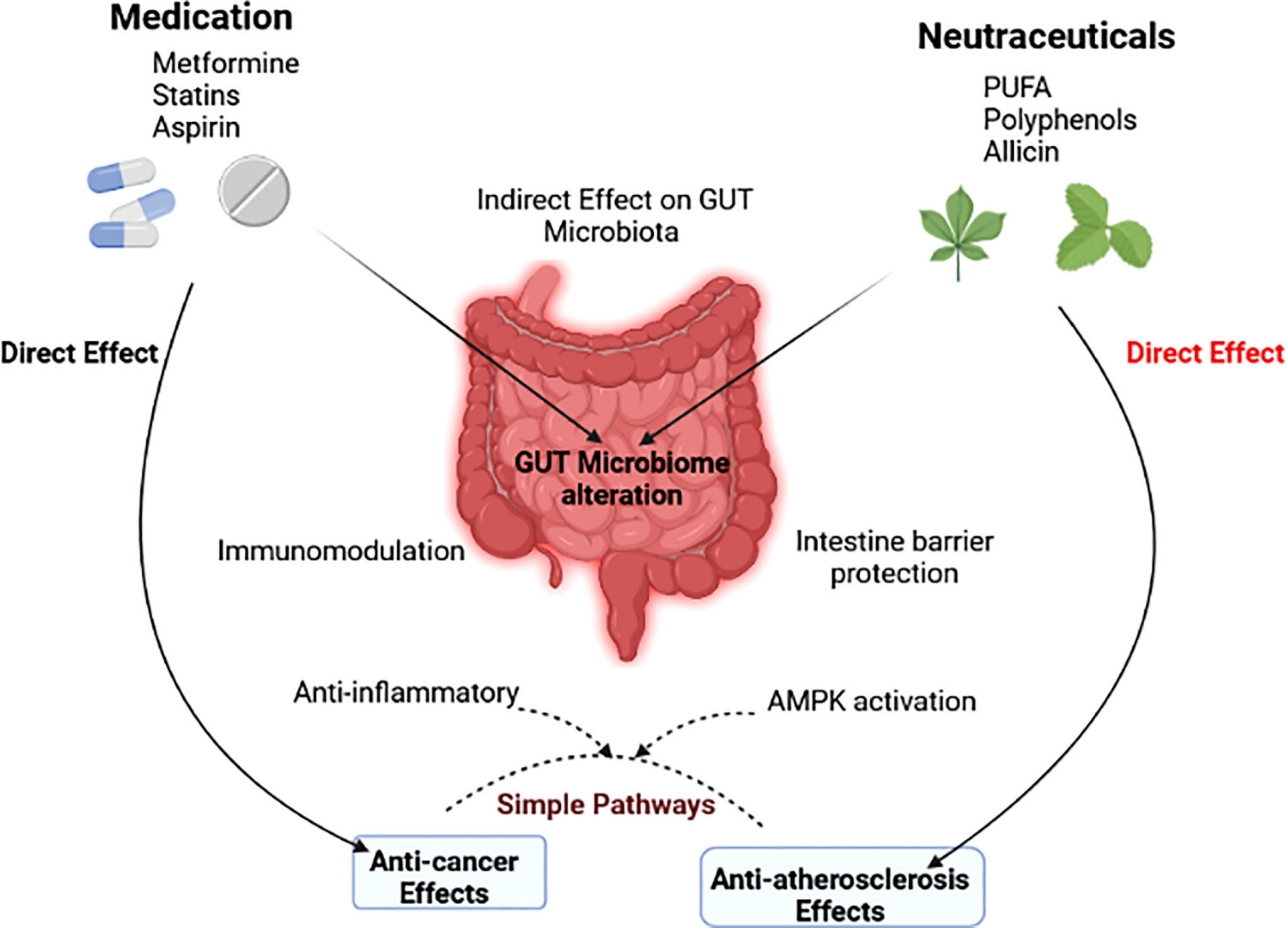
The gut microbiota’s indirect role in modulating the effects of drugs and nutraceuticals is depicted in a diagram (Wu et al., 2021).

Thus, the two most common and deadly human diseases may share similar characteristics that newer treatment techniques could address. Components of the gut microbiota have been demonstrated to have beneficial or harmful impacts on atherosclerosis and cancer. According to growing evidence, the therapeutic benefits of some medications and nutraceuticals are mediated, at least in part, by the gut microbiota. They hence have an indirect influence on the standard mechanisms driving atherosclerosis and cancer (**Figure 5**).

## 11 Patterns of Dysbiosis and Progression of Various Cardiovascular Diseases

Gut dysbiosis is a change in the diversity of the gut microbiota caused by a variety of factors including nutrition, increased stress or inflammatory marker levels, and antibiotic use (Serino et al., 2014). A change in microbial flora could underlie why some people are more susceptible to certain ailments (Carding et al., 2015). Although there is no cause-and-effect relationship between microbial composition and disease propensity, the microbiome is a major contributor in several disease states, an approach that is attracting scientific attention presently (Al Khodor et al., 2017). Gut dysbiosis can disrupt the human body’s homeostatic functioning and have a role in the pathogenesis of a number of metabolic diseases. Dysbiosis, or alterations in microbial composition, is now being related to a variety of non-communicable ailments, such as diabetes (Islam et al., 2022), obesity (Rahman et al., 2022d), cancer (Rahman et al., 2022a), allergic asthma (McKenzie et al., 2017), and others. Several disease states have different microbial compositions or dysbiosis patterns. Emoto et al. (Emoto et al., 2016) recently found a distinct change in microbial composition in individuals with coronary artery disease, with a large rise in *Lactobacillales* (*Firmicutes*) and a decrease in *Bacteroidetes*. Patients with type 2 diabetes had a lower number of *Firmicutes* and a non-significant rise in *Bacteroidetes* and Proteobacteria (Wong, 2014). CVDs, a group of diseases affecting the heart and blood arteries, have been linked to dysbiosis (Gui et al., 2012).

An increasing evidence suggests that CKD patients’ microbiomes are changed. The underlying cause of renal dysfunction, therapeutic interventions prevalent in CKD patients (e.g., antibiotics and immunotherapy), CKD-specific treatment interventions such as iron supplementation and phosphate binders (Dostal et al., 2015), and dietary restrictions (Wong et al., 2014) have all been linked to these shift patterns. Longer intestinal urea concentrations in response to increased blood urea nitrogen (BUN) and increased colonic transit time have long been thought to disrupt the carbohydrate-to-protein balance, resulting in the dysbiosis shown in CKD (Vaziri, 2012; Nazzal et al., 2017). The observations of enhanced urease-producing bacteria in CKD patients and experimental animal experiments backed up these assumptions (Cummings et al., 1979). In mice with medically induced CKD, we recently discovered an elevation in urease-producing bacteria (Chaves et al., 2018).

Despite mounting evidence of a relationship between hypertension and the microbiome, the underlying mechanism for how CKD-induced dysbiosis contributes to hypertension are yet unknown. Short chain fatty acids (SCFAs) are one prospective factor contributing, as they have the ability to regulate blood pressure *via* G protein-coupled receptors (GPCRs) (Yang et al., 2015). Propionate activation of the olfactory receptor78 (Olfr78) in the renal vasculature, for instance, causes renin secretion (Pluznick et al., 2013). However, because it is unknown why SCFA-mediated activation of another GPCR, Gpr41, lowers blood pressure, this SCFA feature remains poorly defined (Vallianou et al., 2020). To better describe the link between circulating SCFAs and systemic blood pressure in CKD, more research is needed in this area. The fact that various G-Protein Coupled Receptors (GPCRs) are located throughout the body and have different SCFA concentration effect profiles demonstrates this (Pluznick, 2016). The hypothesis that inflammation performs a synergistic role in hypertension and CKD development is a second potential link between dysbiosis in CKD and elevated CVD risk. Previous research has found links between low-grade inflammation and CKD development, as well as immunological reliance for angiotensin II-directed hypertension (Kossmann et al., 2013; Karbach et al., 2016).

## 12 Dysbiosis-Associated Diseases

### 12.1 Inflammatory Bowel Disease

Intestinal dysbiosis in inflammatory bowel disease (IBD) patients is associated with decreases in commensal bacteria variety, with the preponderance of the reduction appearing in *Firmicutes* and Bacteroides in the intestinal microflora, the two most prevalent groups in the normal flora. Dysbiosis in IBD, particularly Crohn’s disease (CD), has been linked to an increase in the *Enterobacteriaceae* family, according to some research findings (Nguyen, 2011; Hedin et al., 2014). Five bacterial species are associated with the dysbiosis profile in CD: an increase in *Ruminococcus gnavus*, a reduction in *Faecalibacterium prausnitzii*, *Bifidobacterium adolescentis*, *Dialisterinvisus*, and an unexplained *Clostridium* cluster XIVa (Hedin et al., 2014). Unaffected relatives of CD patients were also found to have a changed intestinal microbiota compared to healthy persons, as well as higher mucin breakdown and epithelial permeability (Shanahan and Bernstein, 2009; Mondot et al., 2011). Because the mucosal membrane in the intestine is the first line of protection against luminal microbiota, this deterioration could be a precursor to dysbiosis and CD. Dysbiosis, according to this reasoning, could be a forerunner to CD. Several probable pathways for the role of dysbiosis in the pathogenesis of IBD are being investigated: One of these processes is a drop in butyrate-producing bacteria accompanied by an increase in sulfate-reducing bacteria (SRBs), which is common in IBD patient dysbiosis (Hedin et al., 2014). In these patients, the dysbiosis in the gut is marked by a significant reduction in *F. prausnitzii*. Butyrate is a source of energy for intestinal epithelial cells and is required to keep the intestinal epithelial barrier from becoming vulnerable to infections. SRB levels have also been observed to be higher in some investigations. Sulfate is metabolized by SRBs into hydrogen sulfide, a hazardous chemical that can prevent butyrate consumption, limit phagocytosis, and kill bacteria (Fava and Danese, 2011). This proposed sequential mechanism for dysbiosis-related IBD indicates that dysbiosis regarded by a reduction in butyrate-producing bacteria and a raise in SRBs results in a lower level of butyrate, which leads to a decrease in epithelial tight junction protein expression and thus continued to increase colonic epithelial permeability, which leads to increased bacterial translocation through the intestinal epithelial cells and lamina propria (Rigottier-Gois, 2013). Malfunctioning phagocytosis impairs the killing of bacteria that reach the lamina propria through the permeable epithelial barrier, which results in excessive Toll-like receptor stimulation, proinflammatory cytokine secretions, and activation of acquired immune responses, all of which significantly raise intestinal inflammatory reactions in genetically susceptible individuals who harbor mutations in the IBD-susceptibility gene(s) (Duboc et al., 2013).

### 12.2 Obesity

Obesity is a metabolic condition characterized by an excess of body fat storage. It is thought to be caused by an energy imbalance, with poor energy expenditures and increased caloric intake. Recent research suggests, however, that obesity is a more complicated condition in both mice and humans, and that it is linked to intestinal dysbiosis (Arslan, 2014). A certain microbial signature appears to be linked to the development of obesity, identical to IBD. In obese people, the bacterial diversity in their intestines is reduced overall (Turnbaugh et al., 2006). Obesity appears to be linked to a modified ratio between *Bacteroidetes* and *Firmicutes* in the majority of research in both humans and animal models, with a reduction in *Bacteroidetes* and an elevation in *Firmicutes*. This ratio has been linked to body weight and fat deposition, indicating that obese people have a higher disproportionate ratio of these bacteria (Hildebrandt et al., 2009). The number of *Bacteroidetes* in the intestinal microbiota appears to be relevant in obesity, as obese people on a calorie-restricted diet lose weight and have a higher ratio of *Bacteroidetes* species in their gut microbiota (Ley et al., 2006a).

### 12.3 Diabetes Mellitus

Insulin-dependent diabetes mellitus (IDDM)-related dysbiosis is marked by an increase in *Bacteroidetes* and *Clostridium*, as well as a reduction in mucin-degrading bacteria such as *Bifidobacteria*, *Lactobacillus*, and *Prevotella* (McLean et al., 2015). In non–insulin-dependent diabetes mellitus (NIDDM) that isn’t linked to obesity, however, dysbiosis is marked by a drop in *Clostridium*, an increase in *Lactobacillus*, and a reduction in *Bacteroidetes*. Both IDDM and NIDDM are linked to a reduction in total microbial diversity, and a drop in butyrate-producing bacteria and *Firmicutes*, as well as a disruption in the intestine epithelial barrier and increased intestinal permeability (Wen et al., 2008; Larsen et al., 2010). Elevated lipopolysaccharide (LPS) translocation and endotoxemia are also reported in NIDDM, which, like obesity, may contribute to reduced inflammation that contributes to the formation of insulin resistance (Wang et al., 2012). It is unknown if the unbalanced microbiota is a causal agent or an outcome of diabetes, as it is in other diseases linked with intestinal dysbiosis; nonetheless, several human and animal model studies indicate that changes in the microbiota may predate the onset of IDDM (Serino et al., 2012). The microbiota structure of nonobese diabetic (NOD) mice with diabetes at weaning age differs from the microbiota composition of NOD mice who do not develop diabetes. The frequency of IDDM was also found to be dependent on the overall bacterial environment in which they were housed. Disease develops in NOD mice in GF settings, but not in mice in specific pathogen-free (SPF) facilities (Nielsen et al., 2014).

### 12.4 Cancer

Patients with CRC have a general dysbiosis pattern, which includes a decrease in butyrate-producing bacteria and a rise in the number of multiple potentially dangerous microorganisms. According to many studies, *Proteobacteria*, *Bifidobacteria*, *Prevotella*, and SCFA production rates have decreased, whilst *Firmicutes*, *Bacteroidetes*, *Enterobacteriaceae*, and *Fusobacteria* have increased (Schulz et al., 2014). Various investigations have also found a rise in two specific bacteria species, *Akkermansia muciniphila* and *Fusobacterium nucleatum*, in CRC tissues (Castellarin et al., 2012). Both of these bacterial species are linked to strong local inflammatory responses, which could be indicative of a high risk of CRC and be involved in inflammation-related disorders. *F. nucleatum* is also linked to an increase in CRC tumors and lymph node cancer. The structure and luminal numbers of dominant microbial species seen in CRC-associated dysbiosis largely depend on pathogenicity and tumor stage/status, according to the findings. Between patient populations with polyps and those with tumors, substantial variations in mucosal and fecal microbial configurations were found, with the most major changes being *Enterobacteriaceae*, which was elevated in the mucosa of patients with tumors compared to those with polyps, and *Bacteroidetes*, which was expanded in CRC tissues with tumor cells compared to those without tumors (Sobhani et al., 2011).

## 13 Conclusions and Future Perspectives

The gut microbiota is a novel topic of research interest with significant applications in human health with respect to the development of innovative diagnostic and therapeutic tools for preventive and curative outcomes. Few studies in humans have investigated the intestinal microbiota’s causative relationships, putative pathways, and metabolites in the context of host disease. Recent molecular and biochemical studies have allowed diverse microorganisms to be detected and categorized and facilitated the analysis of their genomes and metabolites. In particular, genome-scale metabolic models can guide our understanding of the function of individual organisms within the gut microbiota and the role of the microbiota overall since the inter-individual variability of the gut microbiota contributes to different treatment responses and perspectives.

Our mechanistic understanding of how the gut microbiota transforms dietary and endogenous molecules into metabolites shared with peripheral organs and tissues in the host needs substantial expansion. Although the emergence of high-throughput sequencing technologies and bioinformatics over the next decade will inevitably aid in exploring the applicable biological mechanisms, the end of the metabolic pathways sector of the system and the host targets that identify them make up the fascinating puzzle pieces required for the next step in this growing field. Further mechanistic experiments may result in the development of TMAO as a novel biomarker for the primary prevention of CVD. This could lead to a change in the conventional risk factors for CVD and modulation therapies directly targeted at the intestinal microbiota. Metabolic models at the genome scale are statistical descriptions of the metabolic ability of the microbiota that can simulate differences in metabolic system function among different organisms by incorporating knowledge of the gut microbiota’s metabolism.

It is expected that these technological advances will promote the transition from correlation experiments to mechanistic insights, resulting in the development of novel diagnostic tests and therapeutics in the near future. Further research is needed to fully explore the processes underlying host-microbiota interactions to shed light on the biological effects of direct or indirect manipulation of the gut microbiota.

## Author Contributions

MMR, FI, and AAM: Wrote the manuscript; MHR, MSR, MI, and AK: Searched literatures; PS, SM, TE, and MA: Illustrated figures and tables; FW, RI, and TT; Edited the manuscript; AAM and SC: Designed and supervised the review. All authors finally revised and approved for submission of the final version of the manuscript.

## Conflict of Interest

The authors declare that the research was conducted in the absence of any commercial or financial relationships that could be construed as a potential conflict of interest.

## Publisher’s Note

All claims expressed in this article are solely those of the authors and do not necessarily represent those of their affiliated organizations, or those of the publisher, the editors and the reviewers. Any product that may be evaluated in this article, or claim that may be made by its manufacturer, is not guaranteed or endorsed by the publisher.
